# Clinical evaluation of respiration-induced attenuation uncertainties in pulmonary 3D PET/CT

**DOI:** 10.1186/s40658-014-0107-7

**Published:** 2015-02-24

**Authors:** Matthijs F Kruis, Jeroen B van de Kamer, Wouter V Vogel, José SA Belderbos, Jan-Jakob Sonke, Marcel van Herk

**Affiliations:** Department of Radiation Oncology, The Netherlands Cancer Institute, Plesmanlaan 121, 1066 CX Amsterdam, The Netherlands; Department of Nuclear Medicine, The Netherlands Cancer Institute, Plesmanlaan 121, 1066 CX Amsterdam, The Netherlands

## Abstract

**Background:**

In contemporary positron emission tomography (PET)/computed tomography (CT) scanners, PET attenuation correction is performed by means of a CT-based attenuation map. Respiratory motion can however induce offsets between the PET and CT data. Studies have demonstrated that these offsets can cause errors in quantitative PET measures. The purpose of this study is to quantify the effects of respiration-induced CT differences on the attenuation correction of pulmonary 18-fluordeoxyglucose (FDG) 3D PET/CT in a patient population and to investigate contributing factors.

**Methods:**

For 32 lung cancer patients, 3D-CT, 4D-PET and 4D-CT data were acquired. The 4D FDG PET data were attenuation corrected (AC) using a free-breathing 3D-CT (3D-AC), the end-inspiration CT (EI-AC), the end-expiration CT (EE-AC) or phase-by-phase (P-AC). After reconstruction and AC, the 4D-PET data were averaged. In the 4D_avg_ data, we measured maximum tumour standardised uptake value (SUV)_max_ in the tumour, SUV_mean_ in a lung volume of interest (VOI) and average SUV (SUV_mean_) in a muscle VOI. On the 4D-CT, we measured the lung volume differences and CT number changes between inhale and exhale in the lung VOI.

**Results:**

Compared to P-AC, we found −2.3% (range −9.7% to 1.2%) lower tumour SUV_max_ in EI-AC and 2.0% (range −0.9% to 9.5%) higher SUV_max_ in EE-AC. No differences in the muscle SUV were found. The use of 3D-AC led to respiration-induced SUV_max_ differences up to 20% compared to the use of P-AC.

SUV_mean_ differences in the lung VOI between EI-AC and EE-AC correlated to average CT differences in this region (*ρ* = 0.83). SUVmax differences in the tumour correlated to the volume changes of the lungs (*ρ* = −0.55) and the motion amplitude of the tumour (*ρ* = 0.53), both as measured on the 4D-CT.

**Conclusions:**

Respiration-induced CT variations in clinical data can in extreme cases lead to SUV effects larger than 10% on PET attenuation correction. These differences were case specific and correlated to differences in CT number in the lungs.

## Background

Quantitative positron emission tomography (PET) imaging, and ^18^F-fluordeoxyglucose (FDG) in particular, has emerged as a useful tool for comparison between trial outcomes and better response monitoring [1]. At current practice, quantitative PET imaging is still far from being precise. Test-retest reliability for standardised uptake values (SUVs) is about 0.9 [2,3] and measurements of clinical responses on PET are only considered significant and reliable when larger than about 20% to 30% [4]. To make PET more quantitative, uncertainties in image acquisition and processing have to be reduced. Known factors that require standardisation include radiotracer administration, patient weight, blood glucose concentrations, scanner calibration and imaging procedures [2,5].

For thoracic imaging, respiratory motion is a major factor of image degradation, with clinical relevance in PET [6]. Respiratory motion can affect SUV measurement in two distinct ways.

Firstly, respiratory motion will blur the signal, since PET image acquisition takes several minutes per bed position. This causes larger apparent volumes and decreased signal peaks [7].

Secondly, respiration can hinder attenuation correction. The body attenuates the majority of the emitted 511-keV photons and attenuation correction (AC) is therefore essential for accurate quantification of activity concentrations [8]. In older systems, a Germanium-68 transmission scan was used for attenuation correction. Since both scans are acquired in the same duration and experience similar motion blurring, respiratory motion has similar effects on both datasets and no major offset is to be expected between the two [9]. In recent years, x-ray computed tomography (CT) has replaced the transmission scan for AC. Since 3D-CT is acquired in less than a second per slice, each slice depicts the anatomy without motion blurring. However, if the patient breathes during acquisition, each slice will be recorded in a different respiratory position [10]. This will result in anatomical mismatches between PET and CT, which may cause incorrect AC [11,12].

However, not only the tissue position varies during respiration. The lungs are decompressed and compressed to pump air in and out. During compression, the average attenuation coefficient as measured on CT (or CT number) increases within the lungs [13]. This CT number increase will change the attenuation map, with a potential impact on the attenuation correction.

The purpose of this study is to quantify in clinical patient data the combined effects of pulmonary CT differences due to respiration on SUV measurements in 3D FDG PET/CT and identify possible factors causing these differences.

## Methods

### Data acquisition

Between 2010 and 2013, we have acquired PET/CT datasets of 32 lung cancer patients who were candidates for radiotherapy using a combined PET/CT scanner (Gemini TF; Philips Medical Systems, Cleveland, Ohio, USA), eight as part of a study with informed consent and 24 as part of clinical routine. The 4D-PET-CT datasets were rapidly acquired and originally used for the construction of motion compensated mid-position radiotherapy planning scans [14].

Patients received on average 192 (*σ* = 19) MBq of FDG. After injection, the patients rested on average for 72 (range 48 to 105) min. Then, a 3D low-dose CT (3D-CT) whole body scan was acquired (rotation time 0.5 s, detector width 24 mm, 40 mAs, 120 kVp, slice spacing and thickness 3 mm, pitch 0.81). The duration of this scan was around 30 s. Next, a whole body PET emission scan was acquired (2 min per bed position) during which the respiration was monitored using a bellow belt system (Interactive Breath-hold Control System; Mayo Clinic/Medspira, Minneapolis, MN, USA). Finally, a 4D-CT acquisition was performed of the thorax (rotation time 0.5 s, detector width 24 mm, 30 mAs, 90 kVp, slice spacing and thickness 3 mm, pitch 0.085) with respiration monitoring using the same bellow belt system. The duration of this scan was about 120 s. The respiration-monitored PET and CT data were retrospectively reconstructed in 4D with 10 phase bins, using the standard Philips 4D reconstruction software.

The PET image reconstruction was performed with the iterative Philips reconstruction software, using a 3D line-of-response time-of-flight blob-based algorithm using the standard parameter settings (3 iterations, 33 subsets, relaxation parameter: 1.0, transverse image matrix size: 144 × 144 voxels, voxel size: 4 × 4 × 4 mm^3^).

### Attenuation correction

First, we applied phase-by-phase attenuation correction (P-AC), where every 4D-PET respiration phase frame was corrected with the corresponding 4D-CT respiration phase frame. This method was chosen as our reference method. Furthermore, we simulated 3D reconstructions for three situations with different breathing positions on CT, representing current clinical practice and two extremes. We used the free-breathing CT (3D-CT) and extracted the end-inhale (EI-CT) and end-exhale CT (EE-CT) from the 4D-CT. Subsequently, we generated three different 4D reconstructions in which for all phases either the 3D-CT, the EI-CT or the EE-CT was used for AC (3D-AC, EI-AC and EE-AC). All datasets were reconstructed in 4D mode, and the resulting 4D datasets were averaged over the respiratory phases (4D_avg_), to generate comparable 3D reconstructions for 3D-AC, EI-AC and EE-AC.

These methods of generating and averaging 4D data were chosen for a couple of reasons. The 4D-PET data were rapidly acquired with the purpose to be registered and recombined into a MidP scan [7]. The signal per frame was therefore too low for analysis of the individual frames. Furthermore, it is likely that the uptake density will differ between the end-inhale PET and end-exhale PET, since the pulmonary tissue to which the tracer has been distributed is stretched. For this reason, the 4D-PET data were averaged. Because the iterative reconstruction method is non-linear, we also created 4D instead of 3D reconstructions for 3D-AC, EI-AC and EE-AC to avoid reconstruction differences with the P-AC.

To verify the relevance of our methodology for standard 3D clinical data, we have also performed 3D reconstructions of the EI-AC and EE-AC and compared the differences in maximum SUV (SUV_max_) in the tumour to the differences of the 4D_avg_ (data not reported). Both differences showed a high-correlation coefficient (*ρ* = 0.89), indicating our results can be translated to routine 3D data.

### Analyses of SUV differences

The analyses of the PET data were performed with in-house built software on the 4D_avg_-PET data. Per AC method, the tumour was manually identified, and we computed the SUV_max_ in the tumour. The tumour was then automatically delineated using a 42% SUV_max_ threshold, and this delineation was used to assess the tumour volume [15]. Furthermore, we selected two volumes for reference: a volume of interest (VOI) in the centre of the affected lung (away from dense structures where one expects artefacts due to PET-CT mismatch) and a VOI around the subscapularis muscle located at the side of the affected lung. We calculated the average SUV (SUV_mean_) in these two regions for the different AC methods.

A Bland-Altman analysis was used to test the dependency of the SUV difference between EI-AC and EE-AC on the SUV magnitude in the lung VOI. We tested the significance of observed differences in SUV using a two-tailed paired Student's t-test.

For the comparison between the 3D-AC and P-AC, we also investigated the unsigned difference, since we did not expect a strong signed difference between the two.

### Analyses of CT differences

A mask was manually placed around the tumours on the 4D-CT scans. Within this mask, we determined the tumour motion amplitude using local rigid registration [16]. When multiple tumours were present, they were analysed separately. Similar to the tumour motion, we measured the diaphragm motion amplitude of the affected lung, since the diaphragm motion is not dependent on tumour location and therefore a better descriptor of the patient's breathing dynamics.

We determined the average volume difference between inhale and exhale of the affected lungs on the 4D-CT. We did this by manually segmenting the lung volume in the EI-CT and EE-CT and refining this segmentation by applying a threshold under −300 HU. In these lung VOIs, we also calculated the average CT number in EE-CT, EI-CT and 3D-CT.

### Correlations

Finally, we investigated correlations between SUV differences and observed differences in the CT data. We tested for variable dependency with the Pearson's product-moment correlation coefficient. Significance levels of *p* < 0.05 where used.

## Results

### SUV differences

**Figure 1:**
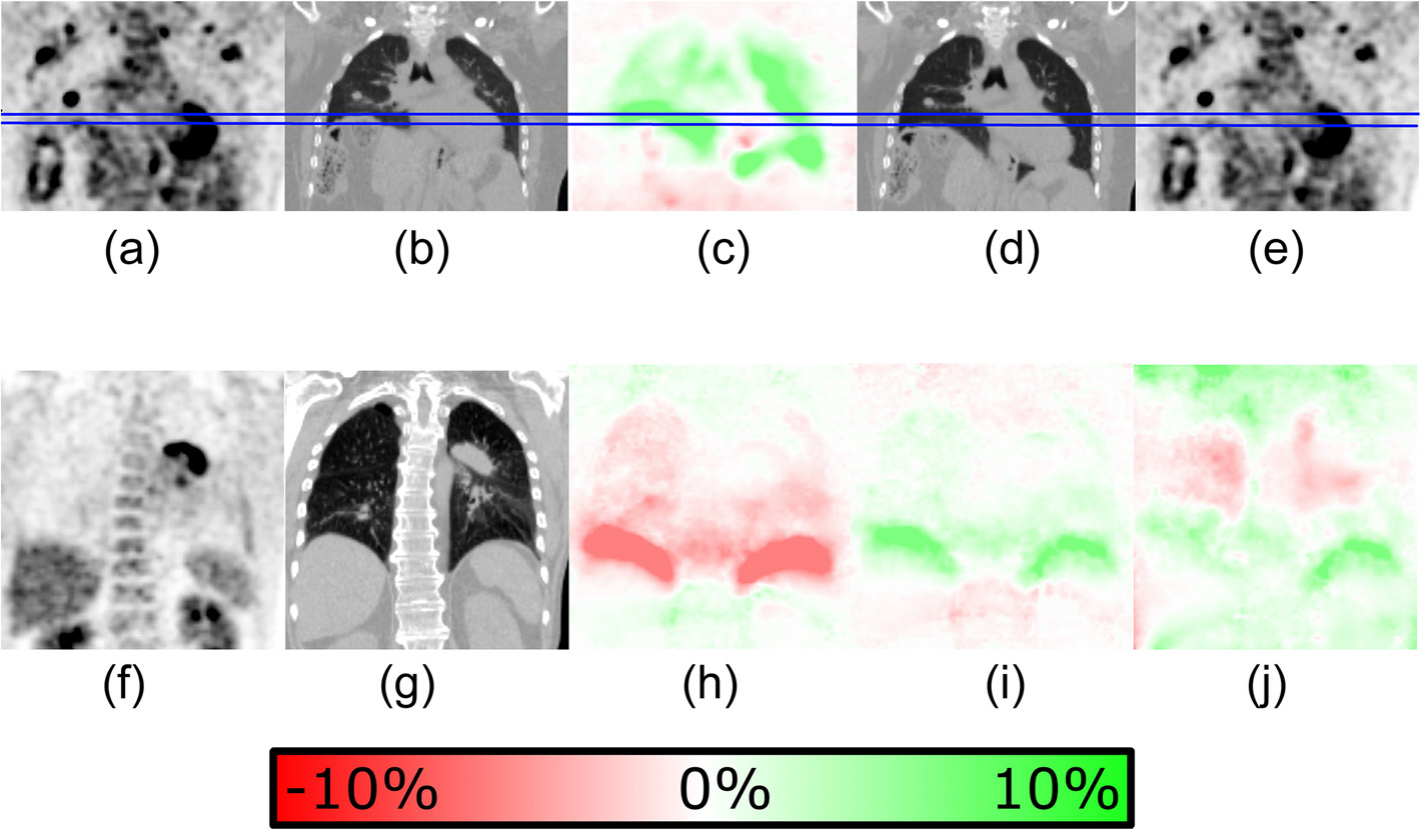
**Two patient examples.** The first row provides the EE-CT **(b)** and EI-CT **(d)** with the EE-AC **(a)** and EI-AC **(e)** of a patient. The blue lines indicate the inspiration and expiration position of the diaphragm. The difference between the two is given, where green represents an increase in the EE-AC PET **(c)**. In the intensely coloured regions, the difference exceeds 10%. Most differences are present within the thoracic cavity, and these differences are mostly consistent. Note that the differences are relative, explaining why contrasts in SUV differences do not correlate as well with local CT contrasts as one would expect in absolute differences. On the bottom row, another patient example is provided. The first two images are the P-AC PET **(f)** and the P-CT **(g)**. Also, the difference between P-AC and EE-AC **(h)** and P-AC and EI-AC is provided **(i)** next to the difference between P-AC and 3D-AC **(j)**. In the images, green indicates an increase in the 3D-AC. It is visible that in **(j)** the differences vary considerably within the thoracic cavity in the last image, while a consistent difference pattern is visible in **(h)** and **(i)**.

Figure 1 illustrates where attenuation correction differences take place. Near the diaphragm, large differences can be observed, as well as inside the mediastinum and the lungs. Outside the thoracic cavity, the differences are hardly visible. The differences between the P-AC and 3D-AC are larger than between EE-AC and EI-AC and show both positive and negative values.

**Figure 2:**
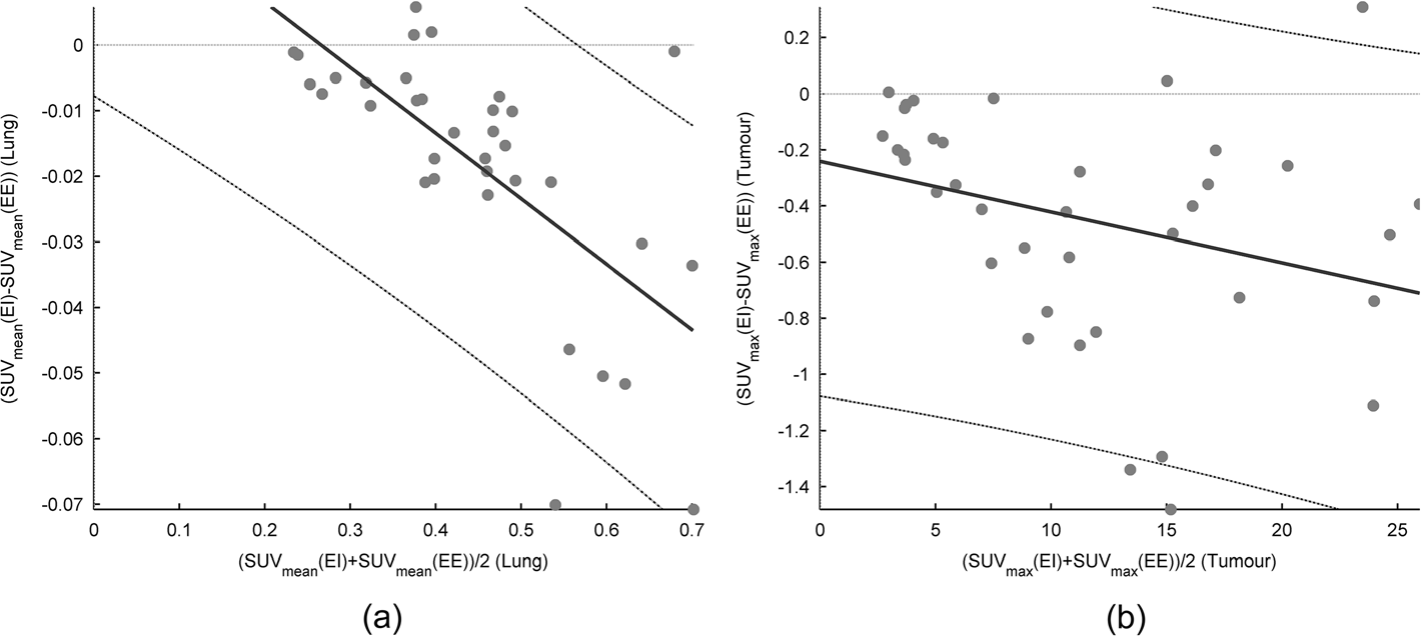
**Bland-Altman plots of the SUV in EI-AC and EE-AC in the lung (a) and the tumour (b).** First-order regression lines indicate a relationship between the SUV intensity and the SUV differences (*y* = 0.027 − 0.10*x*, *p* < 0.0001, with *ρ* = −0.65 for **(a)** and *y* = −0.24 − 0.018*x*, *p* = 0.057, with *ρ* = −0.31 for **(b)**). All regression lines were depicted with 95% confidence intervals.

Bland-Altman comparison between EI-AC and EE-AC of the SUV_mean_ differences in the lung VOI and the SUV_max_ differences in the tumour are provided in Figure 2. The figure shows that there is a proportional bias in the data, especially in the lung VOI, and therefore, we chose to analyse relative differences instead of absolute differences.

**Figure 3:**
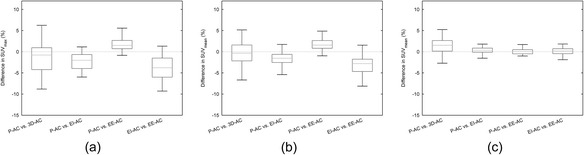
**An overview of differences between the tested AC strategies in three different regions.** Outliers were not displayed in the box plots for clarity (ranges are provided in the text). In both the tumour **(a)** and the lung VOI **(b)**, we found a highly significant SUV increase in EI-AC and a highly significant SUV decrease in EE-AC. In a few samples, where motion was limited, the findings were opposite but within the uncertainty range as illustrated in the muscle data. The average difference in the lungs with the 3D-AC method is the smallest but has the largest spread. In the muscle tissue **(c)**, we found no clear attenuation differences.

In Figure 3, box plots are given of the effects of different attenuation correction strategies on the SUV_max_ in the tumour and SUV_mean_ in lung and muscle in the 4D_avg_-PET data. The SUV_max_ in the tumour on EI-AC was −2.3% (−9.7% to 1.2%) smaller than on the P-AC, while the EE-AC showed an increase of 2.0% (−0.9% to 9.5%). Between EI-AC and EE-AC, the difference was −4.1% (−9.5% to 1.3%). The 3D-AC led to −1.6% (−21.1% to 6.2%) lower SUV_max_ and an average unsigned difference of 3.3% (*σ* = 3.8%). These differences were highly significant according to a paired t-test (*p* < 0.001).

We found a small size difference between the 42% SUV_max_ tumour auto-contours of the EI-AC and EE-AC (1.7% (−3.8% to 17.6%), *p* = 0.015). The 3D-AC however did not give a significant size difference with the P-AC in these tumour auto-contours. The average unsigned size difference in the tumour auto-contours on the PET data between P-AC and 3D-AC was 4.4% (*σ* = 3.9%).

The measured SUV_mean_ differences in the normal lung tissue VOI (Figure 3b) were comparable to the SUV_max_ in the tumour (Figure 3a). The differences with the P-AC were −2.3% (−18.3% to 1.7%) for the EI-CT and 1.3% (−18.7% to 8.8%) for the EE-CT. The latter measure is heavily affected by an outlier of −18.7%. Between EI-AC and EE-AC, the difference was −3.5% (−12.2% to 1.6%). Again, these differences were highly significant (*p* < 0.001). We found no significant SUV_mean_ differences with the 3D-CT method. The average unsigned difference in SUV_mean_ between P-AC and 3D-AC was 2.7% (*σ* = 3.3%) in the lung VOI.

We did not find significant SUV_mean_ differences in the muscle between the EE-AC, EI-AC and the P-AC (Figure 3c). We did however find a small significant (*p* = 0.006) increase of 1.4% (−6.8 to 8.2%) between the P-AC and 3D-AC. The average unsigned difference in SUV_mean_ between P-AC and 3D-AC was 2.2% (*σ* = 1.8%).

### CT differences

In Table 1, an overview of the patient characteristics is provided. The average peak-to-peak tumour motion amplitude in left-right (LR), anterior-posterior (AP) and cranio-caudal (CC) direction was 1.0 (LR), 2.0 (AP) and 4.4 mm (CC), respectively. The average diaphragm motion was 2.1 (LR), 5.4 (AP) and 10.2 mm (CC). This respiratory motion caused the lungs to be 13.1% (*σ* = 5.8%) smaller in end-exhale than in end-inhale, while the lung tissue CT number on the EI-CT was on average 15.6% (σ = 7.0%) lower than on the EE-CT.

**Table 1:** **An overview of patient measurements**

	**Affected lung**	**Tumour SUV**_**max**_	**Tumour size (CC)**	**Motion amplitude tumour (mm)**	**∆CT number (EI-EE) (Lung ROI)**	**∆SUV**_**max**_ **(EI-EE) (Tumour)**	**∆SUV**_**max**_ **(3D-phase) (Tumour)**	**∆SUV**_**mean**_ **(EI-EE) (Lung ROI)**	**∆SUV**_**mean**_ **(3D-phase) (Lung ROI)**	**∆SUV**_**mean**_ **(EI-EE) (Muscle ROI)**	**∆SUV**_**mean**_ **(3D-phase) (Muscle ROI)**
#1	Right	15.1	94.0	1.7	−3.6%	0.3%	−5.4%	−0.5%	0.2%	0.0%	0.2%
#2	Right	18.2	79.4	1.4	−21.8%	−3.9%	−0.3%	−4.1%	−4.0%	0.4%	2.7%
#3	Right	19.9	22.4	2.8	−10.2%	−1.3%	0.6%	−1.8%	4.9%	0.7%	1.3%
#4	Right	11.1	30.9	1.4	−20.2%	−2.4%	−1.0%	−4.1%	−1.3%	1.0%	1.9%
#5	Right	23.4	15.5	3.3	−4.5%	1.3%	−1.7%	−0.6%	1.8%	0.0%	4.5%
#6	Left	2.7	12.2	5.5	−14.0%	−5.4%	1.5%	−2.8%	1.3%	0.1%	3.0%
#7	Right	3.6	1.3	2.0	−14.6%	−5.8%	0.3%	−4.3%	5.2%	−1.5%	0.1%
	Right	11.1	1.0	2.0	-	−7.7%	−8.4%	-	-	-	-
#8	Left	14.2	17.7	7.3	−29.0%	−8.4%	−2.6%	−8.1%	−1.7%	−0.2%	0.8%
#9	Left	14.5	19.8	16.4	−21.0%	−9.3%	−8.8%	−8.0%	−5.7%	−1.4%	8.2%
#10	Right	9.8	2.2	8.6	−12.8%	−7.6%	1.4%	−3.1%	−0.5%	0.2%	0.0%
#11	Right	24.8	33.7	1.7	−25.6%	−2.0%	−1.1%	−5.0%	−0.6%	0.8%	3.6%
#12	Left	24.1	94.1	1.7	−29.7%	−4.5%	−0.4%	−12.2%	−3.6%	0.7%	1.5%
#13	Right	5.9	3.0	6.6	−12.9%	−5.4%	3.0%	−2.8%	2.0%	−1.1%	1.6%
	Right	3.7	8.9	5.7	-	−6.2%	2.8%	-	-	-	-
#14	Left	10.8	25.5	5.2	−17.9%	−5.3%	−3.6%	−4.8%	3.8%	1.5%	−2.7%
#15	Right	13.6	23.4	27.1	−20.6%	−9.5%	−2.5%	−8.0%	−0.3%	−1.4%	−0.4%
#16	Right	12.0	23.0	4.1	−8.9%	−6.9%	4.8%	0.5%	−18.5%	−0.5%	1.1%
#17	Right	7.5	18.8	4.2	−23.3%	−7.8%	−0.5%	−3.1%	1.9%	1.2%	−0.7%
#18	Left	3.0	4.9	1.4	1.3%	0.2%	0.2%	0.4%	1.2%	0.3%	1.9%
#19	Left	3.4	2.9	12.7	−18.2%	−5.8%	−21.1%	−2.2%	−6.7%	1.8%	4.0%
#20	Left	10.7	3.3	6.5	−13.0%	−3.9%	−3.7%	−2.7%	0.7%	−0.6%	1.3%
#21	Left	17.2	6.2	2.0	−15.1%	−1.2%	−1.1%	−4.7%	−0.7%	0.6%	0.9%
#22	Left	7.5	1.9	2.4	−5.7%	−0.2%	1.0%	1.6%	2.1%	1.2%	2.6%
	Right	7.1	1.9	13.4	−19.5%	−5.7%	−4.2%	−1.6%	1.7%	0.8%	2.9%
#23	Left	4.0	2.0	2.2	−11.4%	−0.6%	5.9%	−2.1%	−0.1%	−1.9%	−2.1%
	Right	9.0	2.0	2.4	−12.8%	−6.0%	0.7%	−1.4%	−4.0%	−1.1%	−6.8%
#24	Right	16.2	13.2	8.2	−18.6%	−2.5%	−3.7%	−2.3%	−1.0%	−0.5%	−0.2%
#25	Right	9.5	16.5	2.4	−18.0%	−9.2%	−8.4%	−3.8%	−0.1%	−0.3%	1.9%
#26	Right	5.3	5.6	9.0	−13.9%	−3.2%	6.2%	−2.1%	−2.3%	0.7%	−1.2%
	Right	3.7	2.3	5.1	-	−1.0%	0.4%	-	-	-	-
	Left	4.9	2.4	3.5	−18.1%	−3.2%	0.7%	−3.7%	−0.7%	0.7%	0.9%
#27	Left	16.9	17.5	1.4	−23.2%	−1.9%	−2.7%	−9.6%	1.1%	0.6%	1.6%
#28	Left	26.2	21.5	4.4	−15.4%	−1.5%	−2.8%	−2.0%	0.4%	0.6%	0.5%
#29	Right	23.9	25.0	1.4	−18.3%	−3.0%	−4.8%	−4.6%	2.2%	0.0%	3.0%
#30	Right	5.2	1.8	7.9	−5.4%	−6.7%	−4.1%	−0.1%	−5.5%	0.7%	2.4%
#31	Left	15.4	3.1	5.7	−18.3%	−3.2%	3.0%	−5.3%	−5.6%	−0.3%	5.2%
#32	Left	3.7	4.8	2.0	−11.5%	−1.4%	1.7%	−1.8%	−1.3%	0.0%	1.8%
Mean		11.5	17.5	5.3	−15.6%	−4.1%	−1.6%	−3.5%	−1.0%	0.1%	1.4%
STDEV		7.0	23.4	5.1	7.0%	3.0%	4.8%	3.0%	4.2%	0.9%	2.5%
Min		2.7	1.0	1.4	−29.7%	−9.5%	−21.1%	−12.2%	−18.5%	−1.9%	−6.8%
Max		26.2	94.1	27.1	1.3%	1.3%	6.2%	1.6%	5.2%	1.8%	8.2%
T-test significance						5.56E-08	0.0036	4.1E-06	0.13	0.52	0.0062

An overview with the individual patient measurements. For the SUV values, we also determined the significance level, with the null hypothesis that they were the same, using a two-tailed paired Student's T-test. When two tumours are present in the same lung, the measurements of that lung are only provided once.

### Correlations

**Figure 4:**
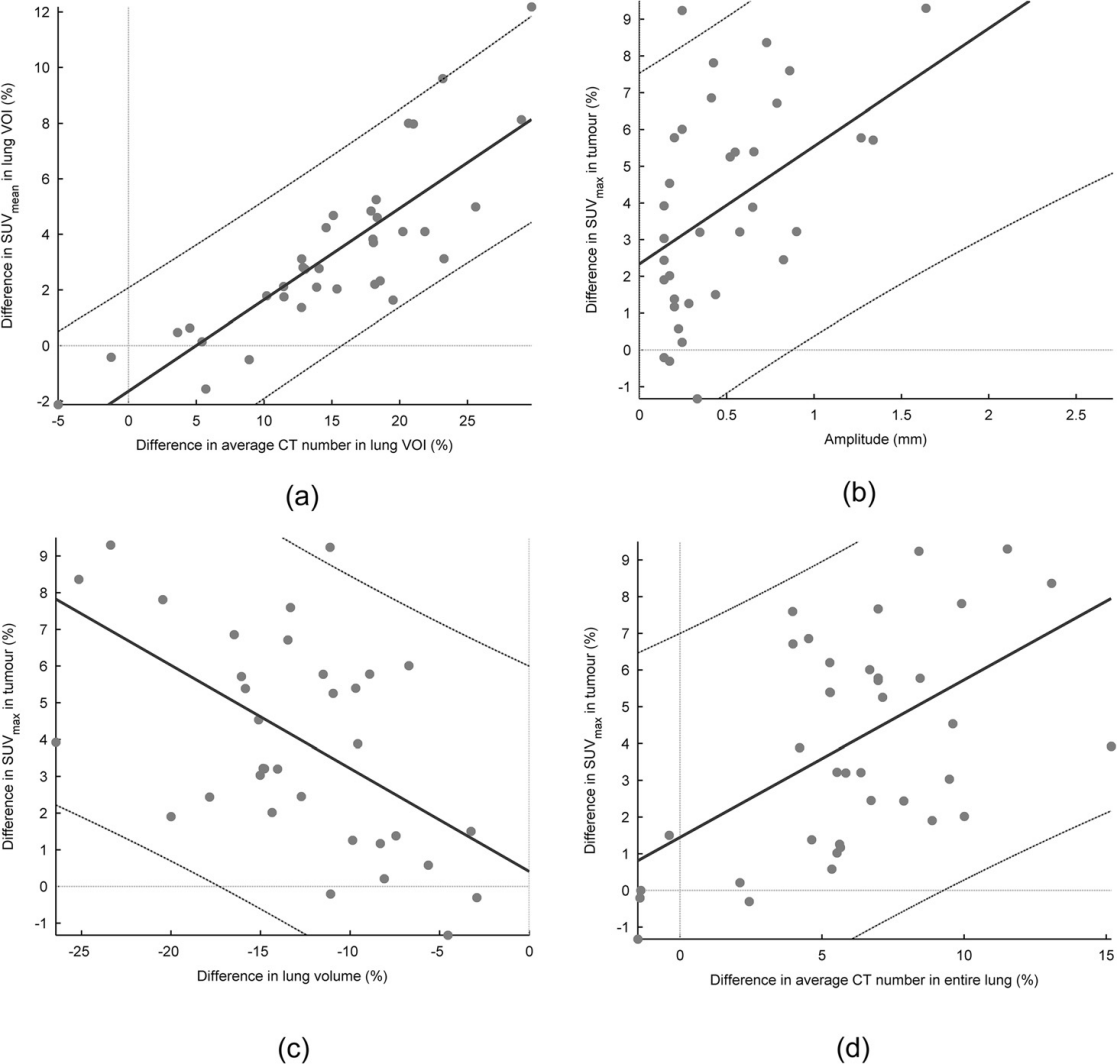
**Scatter plots describing the relationships between SUV differences between EI-AC and EE-AC and some factors.** Scatter plots describing the relationships between SUV differences between EI-AC and EE-AC and some factors, with first-order regression lines and 95% confidence intervals. The relationship between the average CT number and SUV_mean_ in the lung VOIs **(a)** was the strongest (*ρ* = 0.83). We also found a relationship (*ρ* = 0.53) between the SUV_max_ in the tumour and the amplitude **(b)**. This relationship was however mainly predictive for tumours with large amplitudes. The difference in SUV_max_ was also related to the difference in lung volume (*ρ* = −0.55) **(c)** and average CT number in the lungs (*ρ* = 0.50) **(d)**.

We found a linear relation between the average local difference in CT number and difference in SUV_mean_ in the VOI in the lungs (*ρ* = 0.83) between EI-AC and EE-AC. We also found a linear relation between the difference in SUV_max_ in the tumour and the difference in average lung CT number (*ρ* = 0.50) and lung volume (*ρ* = −0.55). Furthermore, a relationship existed between the difference in SUV_max_ and the tumour motion amplitude (*ρ* = 0.53). The latter relationship was especially visible for large tumours. Scatter plots of these relationships are provided in Figure 4.

We did however find no relationship between the differences in SUV_max_ in the tumour and the size of the tumour. Neither did we find a relationship between diaphragm motion amplitude and SUV_mean_ difference in the lung VOI between EI-AC and EE-AC.

## Discussion

In this study, we investigated the effect of respiration on 3D-CT-based attenuation correction of clinical pulmonary PET data, and we have related them to different factors. We have tested the use of the inhale CT, exhale CT and untriggered 3D-CT for attenuation correction and compared this to the respiration-averaged phase-by-phase attenuation correction, which we chose as our gold standard. The SUV_max_ differences between EI-AC and EE-AC in the tumour were on average 4.1% with extremes up to 10%.

The effects of respiration on CT-based attenuation correction have been investigated before in a number of studies [11,12,17], but none of these studies were conducted on fully clinical data, except for [17]. However, the purpose of their study was not to investigate the effects of the use of normal 3D-CT but to test the use of a mid-ventilation CT for attenuation correction.

Nagel et al. [11] describes that mismatches cause a decrease of detected SUV in phantoms and that this decrease is dependent on the size and motion amplitude of the tumour. The motion used for their phantoms (between 25 and 48 mm) was however much larger than measured in this study in patients. The detected tumour [16] and diaphragm motion amplitudes [7,18] were also smaller than reported in other 4D-CT studies. The shallow breathing is likely explained by the resting period that is associated with PET imaging.

We believe that in the lungs (with a less dense background), a tumour mismatch between the modalities will always lead to a decrease in attenuation correction in the tumour [17]. Since EE is closer to the mid-position, this might explain to some extent the difference between EI-AC and EE-AC [19]. However, we also measured an SUV_max_ increase in EE-AC compared to P-AC, which cannot be explained by a position mismatch between PET and CT. A more probable explanation is that, as the diaphragm rises, the lung volume decreases, resulting in denser lung tissue on EE-CT and hereby changing the attenuation characteristics. We found a strong relationship (*ρ* = 0.83) between the average CT difference in the lungs between EI-CT and EE-CT and the change in SUV_mean_ in EI-AC and EE-AC in the lungs. Note that this effect should be smaller for chest breathing than for diaphragmatic breathing; as for chest breathing, the lower CT density is, to a larger extent, compensated for by an increased path length in the direction of the detector.

We also found a relationship between the difference in SUV_max_ in the tumour and both the CT difference (*ρ* = 0.50) and volume difference (*ρ* = −0.55) of the entire surrounding lung, indicating that the difference in lung density has an effect on the attenuation of the PET signal emitting from the tumour. We also found a relationship of these differences with the amplitude (*ρ* = 0.53), particularly for tumours with a large amplitude. This relationship could be caused by a correlation between local amplitude and local density differences. It is also very likely that for tumours with a large amplitude or a small size, mismatches will have an effect on SUV differences. The effects of a mismatch on SUV_max_ will however only be apparent when the tumour amplitude is in the range of the dimensions of the tumour, since mismatch needs to be so severe that the SUV peak is placed outside the tumour volume on the CT.

The reported findings are visible in the resulting PET data. Figure 1 provides examples of the difference between the use of the inhale or the exhale CT scan. The image shows that SUV differences are not restricted to the lungs but affect a large extent of tissue in the thoracic cavity.

As expected, we did not find any respiratory-related attenuation differences in muscle, since no respiratory motion or compression is expected there. We did however find a small, but significant, SUV increase in the 3D-AC, in comparison to the P-AC in the muscle. The reason for this bias did not become apparent. We suspect that posture differences between the two CT acquisitions may play a role.

Figure 1 demonstrates that the local differences in the body between P-AC and 3D-AC are not homogeneous but vary between differences found in EE-AC and EI-AC. This can be explained by the fact that the respiratory phase in which the individual slices were measured in a free breathing scan, since a typical whole body scan takes about 30 s. The scan is therefore composed of slices from about seven different breathing cycles. The differences with the P-AC will therefore also vary, depending on the respiration phase in which the scan locally was acquired. In the example in Figure 1, 3D-AC SUVs are lower in the upper lung (implying exhale), while the SUVs are higher in the lower lung and around the diaphragm (implying inhale).

We found only a small significant tumour size difference (1.7%) between EI-AC and EE-AC and no size difference between P-AC and 3D-AC. This is most likely explained by the fact that the changes in SUV_max_ of the tumour follow the changes in the rest of the lungs. The relative contrast between the two will therefore not change, and therefore, the apparent size and visibility of lesions are therefore hardly affected. It is thus unlikely that the reported effects will have a major impact on the diagnostic value of PET imaging and patient management.

For quantitative PET imaging, however, the differences are of more importance. Clinical response measurements are only considered significant when larger than 20% to 30% [5]. These uncertainties are so large, because clinical response measurements need to be distinguished from measurement error outliers. To make clinical response measurements more accurate, it is therefore important to reduce measurement outliers. We found an average unsigned SUV_max_ difference of 3.3% (*σ* = 3.8%) in the tumour between the 3D-AC and P-AC. On average, these differences are modest, but extreme cases (up to 21.1%, while differences of 10% are very common) will have strong effects on the test-retest variability.

In this work, we compared our measurements against the P-AC. Although we did not prove that P-AC is more correct than other 3D-AC, EE-AC or EI-AC, it is likely that this is the case, since PET and CT frames correspond better. Whether the quantitative improvements are worth the extra dose that is associated with 4D-CT is debatable and dependent on the purpose of the scan. The described differences in AC will not have a large effect on the diagnostic value of PET but more on quantitative measurements. Furthermore, 4D imaging has other advantages, in terms of image sharpness [14] and reduction of motion artefacts, and is especially useful in radiotherapy [16] to improve target definition and measure motion amplitudes. The improvements in attenuation correction will add to that.

Another way to deal with these discrepancies would be to acquire controlled breath-hold PET and CT data. Studies have been demonstrated that patients can be trained to reposition their breath-hold quite well. This however takes some training and may introduce additional offsets when not performed well [20].

## Conclusions

We have quantified the effects of respiration-induced CT differences on attenuation correction in combined PET/CT of the lungs. These effects are modest on average but can be substantial in extreme cases; the overall SUV_max_ difference in the tumour between the use of phase-by-phase attenuation correction or a 3D-CT dataset in the tumour ranged up to 20%. Between the use of EI-AC and EE-AC, we found on average significant tumour SUV_max_ differences of 4.1% with extremes up to 10%. The differences correlated with differences in average CT number in the lungs. SUV_max_ differences in the tumour correlated with both the density of the surrounding lungs and the tumour motion amplitude, especially for large amplitudes. Strategies to minimise respiration offsets between PET and attenuation CT are therefore important to reduce SUV variability.
